# Lymphedema Leads to Fat Deposition in Muscle and Decreased Muscle/Water Volume After Liposuction: A Magnetic Resonance Imaging Study

**DOI:** 10.1089/lrb.2017.0042

**Published:** 2018-04-01

**Authors:** Mattias Hoffner, Pernilla Peterson, Sven Månsson, Håkan Brorson

**Affiliations:** ^1^ Department of Clinical Sciences, Lund University, Malmö, Sweden.; ^2^ Department of Surgery, Blekinge Hospital, Karlskrona, Sweden.; ^3^ Medical Radiation Physics, Department of Translational Medicine, Lund University, Skåne University Hospital, Malmö, Sweden.; ^4^ Department of Plastic and Reconstructive Surgery, Skåne University Hospital, Malmö, Sweden.

**Keywords:** lymphedema, liposuction, adipose tissue, fat, MRI, water–fat imaging

## Abstract

***Background:*** Lymphedema leads to adipose tissue deposition. Water–fat magnetic resonance imaging (MRI) can quantify and localize fat and water. The presence of excess fat and excess water/muscle in the subfascial compartment of the lymphedematous limb has not been investigated before. The aim of this study was to investigate epifascial and subfascial fat and water contents in patients with chronic lymphedema before and after liposuction.

***Methods and Results:*** Seven patients with arm lymphedema and six with leg lymphedema were operated on. The limbs were examined with water–fat MRI before liposuction (baseline) and at five time points. Complete reduction of the excess limb volumes was achieved. The excess epifascial fat was evident in the edematous limbs and a drop was seen following surgery. There were differences in excess water at all time points. At 1 year there was a decrease in excess water. Excess subfascial fat was seen in the edematous limbs at all time points. Subfascial excess water/muscle did not show any differences after surgery. However, starting from 3 months there was less subfascial water/muscle compared with baseline.

***Conclusions:*** Subfascial fat in the lymphedematous limbs did not change. In contrast, the water in the subfascial compartment was reduced over time, which may represent a decrease of muscle volume after treatment due to less mechanical load after liposuction. Using water–fat MRI-based fat quantification, the fat and water contents may be quantified and localized in the various compartments in lymphedema.

## Introduction

Lymphedema is a chronic, complex, and multifaceted condition that has major physical, psychological, and social implications for the quality of life of patients suffering from it.^1,2^ Any disruption of the lymph flow due to disease or iatrogenic damage (surgery, radiotherapy, or trauma) can result in failure to transport lymph back to the blood circulation, resulting in a lymphedema.

Stagnation of the lymph fluid circulation will, if left untreated, enhance tissue changes such as excess adipose tissue and excess fibrosis deposition in the affected limb. Adipose tissue deposition in lymphedema probably occurs because the fat cell is not simply a container of fat, but is an endocrine organ and a cytokine-activated cell, and chronic inflammation plays a role here.^3^ Earlier reports have attributed the excess adipose tissue to a slow lymph flow accelerating lipogenesis and fat deposition.^4^ This process is enhanced by the transformation of macrophages into adipocytes.^5,6^ Subsequently, subcutaneous lymphedema becomes firm due to pinocytosis of white blood cells and activation of fibroblasts, which will increase the connective tissue component of the lymphedema.^7,8^ More recent research focuses on inflammation and upregulation of fat differentiation markers as an alternative course of adipose tissue deposition.^9–12^ Furthermore, lymph fluid stasis drives adipose-derived stem cells toward adipogenic differentiation.^13^

Lymphedema remains a significant clinical problem with 20% of women developing the condition following treatment for breast cancer.^14,15^ In spite of recent sentinel node dissection in breast cancer treatment, still 5.6% are affected.^15^ In addition, up to one third of women report leg lymphedema following gynecological cancer treatment.^16^ Patients with lymphedema are susceptible to erysipelas and the concomitant inflammation may increase adipose tissue deposition.^17^

Since lymphedema evolves from a soft pitting state to an irreversible fatty and fibrotic state, which also may show pitting, it is important to define the different stages in lymphedema. One definition has been introduced by the International Society of Lymphology: “Stage 0 (or Ia) which refers to a latent or sub-clinical condition where swelling is not yet evident despite impaired lymph transport, subtle changes in tissue fluid/composition, and changes in subjective symptoms. It may exist months or years before overt edema occurs (Stages I–III). Stage I represents an early accumulation of fluid that is relatively high in protein content (e.g., in comparison with ‘venous’ edema), which subsides with limb elevation. Pitting may occur. An increase in various proliferating cells may also be seen. Stage II signifies that limb elevation alone rarely reduces tissue swelling and pitting is manifest. Late in Stage II, the limb may or may not pit as excess fat and fibrosis supervenes. Stage III encompasses lymphostatic elephantiasis where pitting can be absent and trophic skin changes such as acanthosis, further deposition of fat and fibrosis, and warty overgrowths have developed.”^18^

A lymphedema, diagnosed at an early stage, is treated conservatively (i.e., combined physical therapy using daily bandaging until pitting disappears, followed by measurement for made-to-measure compression garments) to reduce the edema.^3,19^ For a late stage lymphedema this treatment will be unsuccessful because it is in an irreversible state with adipose tissue deposition and only surgical treatment will reduce the excess volume completely.^20,21^

Depending on the lymphedema stage, the lymphedema will not only have a varying subjective and objective impact on patients' quality of life, but also an impact on choosing the appropriate treatment method (i.e., conservative treatment, microsurgical procedures, liposuction in combination with conservative treatment). To obtain accurate information concerning the relations of the lymphedema components (excess fat hypertrophy, edema) there is a need for reliable noninvasive diagnostic imaging techniques since no consensus on their use exists.^22^

There are different imaging techniques, each with their own advantages and disadvantages, giving insights into the tissue changes due to lymphedema^22^ (i.e., ultrasonography, computed tomography, dual-energy X-ray absorptiometry, and magnetic resonance imaging [MRI]), but they usually focus on the dermal and subcutaneous levels.

The presence of excess epifascial fat has previously been investigated using computed tomography and dual-energy X-ray absorptiometry.^20,21^ However, these techniques are not suitable for localized measurement of the much lower fat concentrations expected in the subfascial compartment. Using water–fat MRI for fat quantification, the fat and water contents may be both quantified and localized.^23^ This method separates the acquired MRI images into water and fat images,^24,25^ which are used to quantify the percentage fat fraction (FF). After consideration and correction of various sources of bias, the approach is robust^26^ and has been validated against independent measurements.^27,28^ This method has mainly been used for liver and muscle applications.^23,29^

The potential presence of excess fat and excess water/muscle in the subfascial compartment before and after liposuction of the lymphedematous limb has not yet been investigated.

The aim of this study was to use water–fat MRI to investigate epifascial and subfascial fat and water contents in patients with chronic arm or leg lymphedema before, and at five time points after liposuction.

## Materials and Methods

### Study design and subjects

Seven patients with arm lymphedema and six patients with leg lymphedema who were scheduled to receive liposuction of the edematous limb participated in this study (Table 1). All arm lymphedema patients occurred following breast cancer treatment. Of the six leg lymphedema patients, three had been treated for cancer (two with uterus cancer and one with synovial sarcoma in the groin) and three had primary lymphedema (one male and two females). All patients had been treated conservatively before liposuction and were wearing compression garments daily. Thus, there was no or minimal pitting when performing the pitting test.^3,19,30^ In addition, 10 healthy volunteers were recruited as controls (median age 30 years; first and third quartiles [1q–3q] 28–44 ; 7 right-handed, 3 left-handed).

**Table 1.** Characteristics of the Study Population (Median [1q-3q])

	*Arms*	*Legs*
Number of patients	7	6
Excess volume preoperatively (mL)	1345 (865 to 1548)	3733 (2920 to 5618)
Excess volume postoperatively at 1 year (mL)	−180 (−374 to 243)	−430 (−513 to −74)
Age at liposuction (years)	62 (60 to 71)	35 (31 to 52)
Duration of lymphedema (years)	6.0 (3.5 to 8.5)	9.0 (4.5 to 14)

The study was approved by the Ethics of Human Investigation Committee at Lund University (LU 2006/503 and 2011/45) and informed consent was obtained from all subjects. The procedures were in accordance with the Helsinki Declaration of 2013.

### Plethysmography

Limb volumes were measured with water plethysmography at six time points (baseline, 2 weeks, 1 month, 3 months, 6 months, and 1 year).^19,30,31^ For plethysmography, all patients were measured, except for one at 1 year due to recurrence of the breast cancer. The healthy controls were not measured.

### Magnetic resonance imaging

The healthy and edematous limbs of patients were examined with water–fat MRI before liposuction (baseline) and at five time points (4 days, 1 month, 3 months, 6 months, and 1 year) after liposuction.

At 6 months, 3 patients could not be measured due to technical problems. At 1 year, 12 patients were measured and 1 could not be measured because of recurrence of the breast cancer. Both forearms of the healthy volunteers were examined with MRI at one time point, and their right forearm was imaged a second time with repositioning to evaluate the methods repeatability. This investigation has previously been described^29^ and resulted in a high repeatability, where the 95% confidence interval of repeated measures of the percentage fat content within the subfascial compartment was within ±0.4%. Only the first of the two measurements of the right arm is used in this work.

### MRI protocol

All MRI examinations were conducted with a 1.5 T MRI scanner (MAGNETOM Sonata; Siemens Healthcare) using a small flex coil (arms) and large flex coil (legs). Arms were imaged separately, whereas both legs were imaged together. Imaging was centered 10 cm distally of the humeral epicondyle (arms), or 16 cm distally of the femoral condyle (legs).

A multiecho gradient-echo sequence was used to image three 5-mm slices with 5-mm interslice gap and 1.6 × 1.6 mm^2^ resolution. Eight echo times (first TE = 1.83 ms, interecho time = 2.47 ms) were acquired with TR = 600, flip angle 10°, bandwidth = 815 Hz/pixel, field of view = 400 × 200 mm^2^, number of averages = 4, and acquisition time = 5 minutes and 6 seconds. The images were reconstructed offline in MATLAB (R2013a; MathWorks, Natick, MA) to separate water and fat images using a combination of magnitude and complex data^32^ with correction for B0 inhomogeneity, a single T2*, and multiple fat peaks.^33^ From the separated water and fat images, the FF was calculated in each image voxel as FF = *F*/(*F* + *W*). The water fraction (WF) was estimated as WF = *W*/(*W* + *F*).

In each image slice, polygonal regions of interest (ROI) were manually drawn covering the epifascial and subfascial compartments (excluding bone and bone marrow). In each ROI, the total water and fat volumes were calculated as the voxel volume multiplied by the sum of the WF or FF of each voxel within that ROI. Thus, for each limb of each patient four measures were obtained within a 15-mm section of the limb: the subfascial water volume, the subfascial fat volume, the epifascial water volume, and the epifascial fat volume. Note that the subfascial water volume includes both edematous fluid and muscle tissue.

### Statistical analyses

All statistical testing were carried out in MATLAB (R2013a; MathWorks). The excess volume was defined as the difference between the lymphedematous limb and the healthy limb in the patient group, and the difference between dominant and the nondominant limb in the volunteer group.

**FIG. 1.**
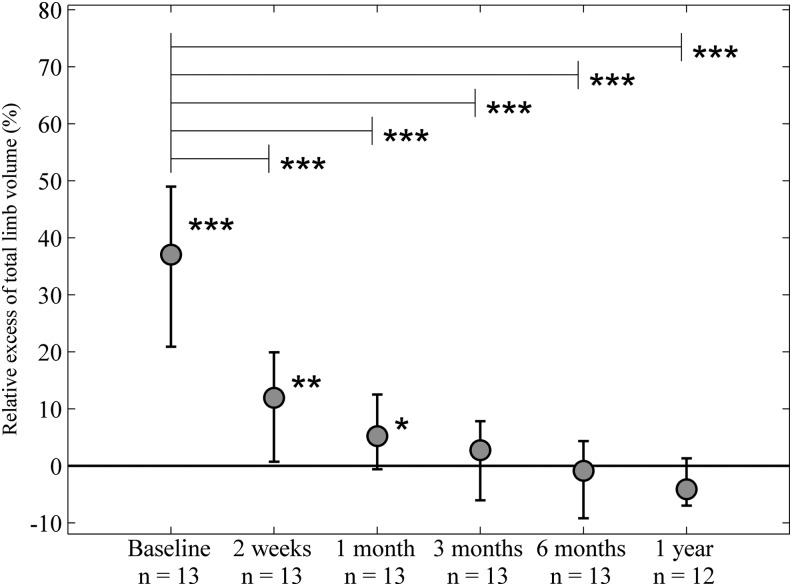
Relative excess total limb volume (%). A significantly larger excess epifascial fat volume was seen in the edematous limbs at baseline and at 2 weeks and 1 month after surgery. No significant difference between limbs was seen at 3 months, 6 months, and 1 year after surgery (*lower asterisks*). A significant drop in total excess limb volumes remained for all postoperative time points compared with baseline (*upper asterisks*). *p < 0.05, **p < 0.01, ***p < 0.001.

**FIG. 2.**
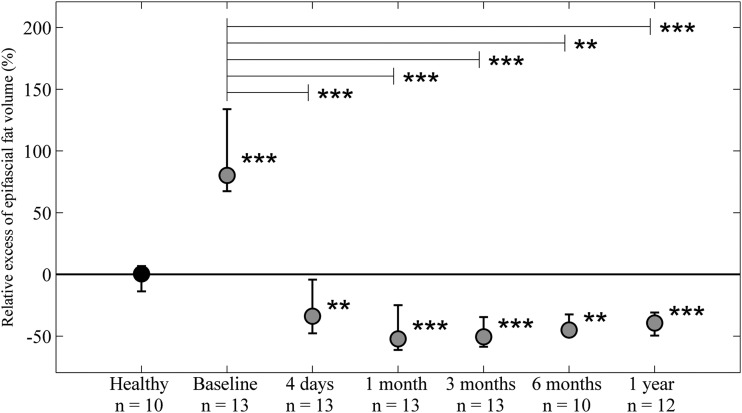
Relative excess epifascial fat volume (%). A significantly larger excess epifascial fat volume was seen in the edematous limbs at baseline. Following surgery a significantly lower excess epifascial fat volume was noted in the edematous limbs at all time points (*lower asterisks*). A significant drop in epifascial excess fat volume remained for all postoperative time points compared with baseline (*upper asterisks*). No difference in the fat volume was seen between the extremities in healthy controls. **p < 0.01, ***p < 0.001.

**FIG. 3.**
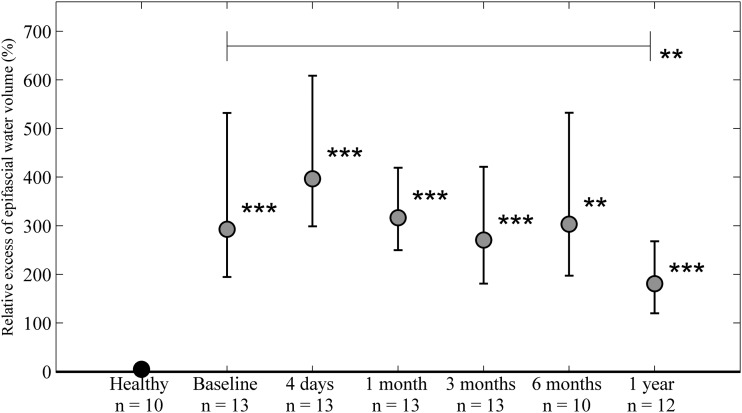
Relative excess epifascial water volume (%). A significantly larger excess epifascial water volume was seen in the edematous limbs at baseline and at all time points after surgery (*lower asterisks*). No significant change in the excess water volume was seen compared with baseline until after 1 year, where a smaller excess epifascial water volume was seen (*upper asterisks*). No difference in the water volume was seen between the extremities in healthy controls. **p < 0.01, ***p < 0.001.

**FIG. 4.**
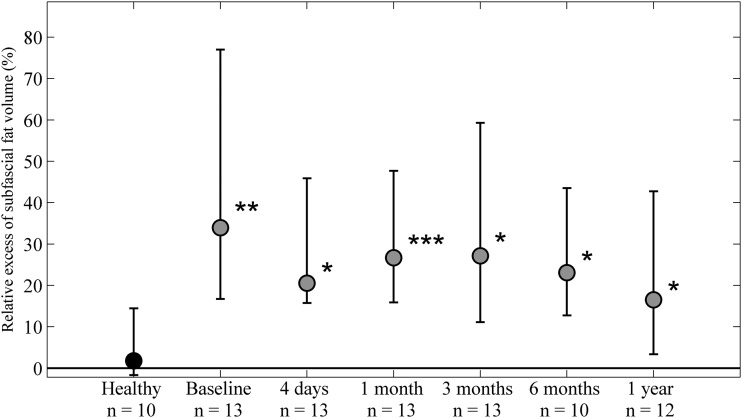
Relative excess subfascial fat volume (%). A significantly larger excess volume of subfascial fat was seen in the edematous limbs at all time points. No significant change in the excess subfascial fat was seen compared with baseline at any time. No difference in the subfascial fat volume was seen between the extremities in healthy controls. *p < 0.05, **p < 0.01, ***p < 0.001.

**FIG. 5.**
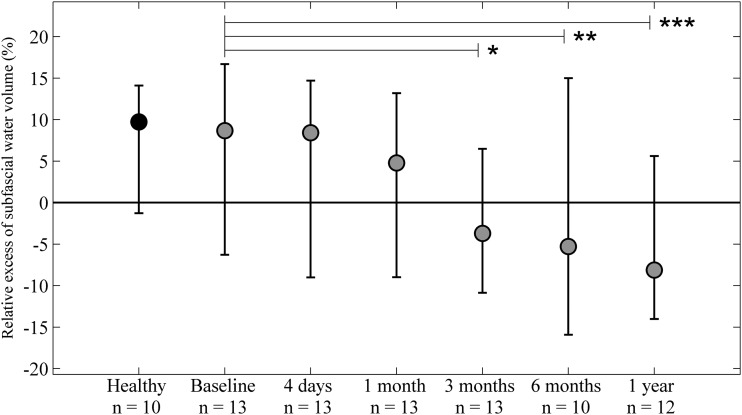
Relative excess subfascial water/muscle volume (%). No significant difference in relative subfascial excess water/muscle volume between the limbs was detected at any time point. Following surgery a significant drop in the subfascial excess water/muscle volume was seen at 3 months, 6 months, and 1 year compared with baseline. No difference in excess water/muscle volume was seen between the extremities in healthy controls. *p < 0.05, **p < 0.01, ***p < 0.001.

At each time point, the relative excess volume (percent) was estimated as (volume edematous limb − volume healthy limb)/volume healthy limb for patients and as (volume dominant − volume nondominant)/volume nondominant arm for healthy controls. These values were compared between baseline and each postoperative time point using a Wilcoxon signed-rank test. Significant changes from baseline to postoperative time points are shown with upper asterisks in Figures 1–5.

Moreover, the Wilcoxon signed-rank test was also used to compare the edematous and healthy side (patients) at each time point. For volunteers, the dominant and nondominant arm was measured once. Significant differences between limbs are shown with lower asterisks in Figures 1–5. Medians (1q–3q) were calculated for outcomes in Table 1. The outcome of these tests was considered to show exploratory results, and therefore nominal *p*-values are presented without any adjustment for multiple comparisons. For all tests, *p* < 0.05 was considered a significant result.

## Results

### Limb volume reduction

Complete reduction of the excess limb volumes was achieved after liposuction in both arm and leg lymphedema patients at 1 year after surgery (Fig. 1). At baseline, significant excess limb volumes were seen in the edematous limbs. After liposuction, a drop in the excess limb volume was seen in the lymphedematous extremities resulting in a significantly lower excess limb volume at 2 weeks and 1 month postoperatively compared with baseline. At 3 months, 6 months, and 1 year no significant difference between the limbs was seen due to a normalization of the volume of the affected limbs (lower asterisks in Fig. 1). Consequently, a significantly lower excess limb volume remained for all postoperative time points compared with baseline (upper asterisks in Fig. 1).

### Epifascial excess fat volume

At baseline, a significant excess epifascial fat volume was evident in the edematous limbs (Fig. 2). As liposuction was performed subcutaneously between the skin and the muscle fascia, a significant drop in the excess fat volume was seen in the lymphedematous extremities resulting in a significantly lower excess epifascial fat volume for all time points following surgery (lower asterisks in Fig. 2). Consequently, a significantly lower excess epifascial fat volume remained for all postoperative time points compared with baseline (upper asterisks in Fig. 2). No difference in the epifascial excess fat volume was seen between the extremities in healthy controls.

### Epifascial excess water volume

Epifascial excess water volume represents edema fluid in the epifascial compartment. During follow-up, there were significant differences between limbs in excess volumes at all time points (lower asterisks in Fig. 3) indicating that there was still a fluid component in the compartment. At 1 year there was a significant decrease compared with baseline in excess water volume (upper asterisks in Fig. 3), which may have been caused by efficient use of compression garments. No difference in the epifascial excess water volume was seen between the extremities in healthy controls.

### Subfascial excess fat volume

A significant excess subfascial fat volume was seen in the edematous limbs at all time points (Fig. 4). For obvious reasons, liposuction is not performed in the subfascial compartment and therefore a significant difference to baseline is not displayed at any time point. No difference in the subfascial excess fat volume was seen between the extremities in healthy controls.

### Subfascial excess water/muscle volume

During follow-up, there were no significant differences between the lymphedematous and normal extremities at any time point (Fig. 5). However, starting from 3 months there was a significantly smaller subfascial water/muscle volume compared with baseline. No difference in the subfascial excess water/muscle volume was seen between the extremities in healthy controls.

## Discussion

Using water–fat MRI, the fat and water contents may be both quantified and localized in the various compartments in the lymphedematous limb. In this study, we show how excess fat and water/muscle volumes in lymphedema change over time in two different compartments after liposuction. Water–fat MRI cannot differentiate between muscle tissue and water using the technique presented in this article; thus, the measured excess subfascial water volume represents both edematous fluid and muscle tissue.

Healthy controls were examined to investigate any differences between the dominant and nondominant sides in a nonlymphedematous group in the various compartments. As can be seen in the figures, no such differences were found.

We noticed, starting from 3 months, a significantly smaller subfascial water volume compared with baseline (Fig. 5). The reason for this is unclear, but is most likely due to a switch from a hypertrophied muscle cell state to a normal muscle cell state caused by less mechanical load from the heavy lymphedematous extremity.^21,34^

Our finding of increased volume of subfascial fat in arm and leg lymphedema using a robust and validated MRI technique has not been reported in the literature, and might in the future help us acquire additional information about the evolution and symptoms connected to lymphedema.

Diagnosis of lymphedema is determined by patient history, subjective/objective symptoms, and measurement of volume differences between the affected and nonaffected limbs. In early stages of lymphedema, neither volume measurements nor physical examination will provide detailed information about the stage of lymphedema, which might have a negative impact on choosing an appropriate treatment modality.

Although previous research done on detecting lymphedema at an early stage and at a superficial localization with tissue dielectric constant (TDC) and local or locoregional bioimpedance spectroscopy (BIS), there are discrepancies between them in assessing lymphedema. These are due to different measurement techniques and assessed tissue water components. Currently there is no scientific evidence on whether the diagnostic power of these local/locoregional BIS measurements would be better than that of the conventional and widely used BIS technique.^35^ Further development of both of these measurement techniques and correlation of results with imaging are important before translation into routine clinical practice.^36^ On the other hand, water–fat MRI not only detects superficial tissue changes, but also changes in the whole composition of the extremity. Thus, water–fat MRI can possibly be used as a supplement to BIS and TDC in standardizing these methods, and in addition to analyze deep tissue changes before and after treatment.

### Clinical aspects and the need for further investigations

Fat quantification using water–fat MRI is a well-established technique that has been used to gain insights into the pathophysiology of metabolic diseases, including obesity, metabolic syndrome, or type 2 diabetes. The perspectives are promising, but this field of research still requires further investigations before clinical application.^37^

Fat serves as an energy reservoir, but more importantly, since the discovery of leptin^38^ it has also been shown to play an important endocrine role influencing a variety of physiological and pathological processes, including immunity and inflammation.^39^

Fat accumulation and fibrosis in the epifascial compartment are clinical hallmarks of lymphedema. In 2012, Zampell et al. showed that lymphatic obstruction in an animal model led to a significant fat and collagen deposition.^10^ In addition, their findings of fat hypertrophy and an increased number of adipocytes suggest that the process of fat accumulation, in response to lymphatic fluid stasis, mimics the events that occur in obesity in general.^40^

In 2009, Boettcher et al. showed that the amount of intermuscular adipose tissue (IMAT) is higher in males compared with females and, furthermore, it increases significantly with age. Compared with other fat compartments, the amount of IMAT was found to be a good predictor for insulin sensitivity, with a correlation coefficient almost equal to that of visceral adipose tissue (VAT). It seems that IMAT and VAT share an analogous pattern in distribution and association with insulin sensitivity. Excessive accumulation of IMAT (subfascial fat) is closely related to insulin resistance,^41,42^ and may also be linked to age,^43^ inflammation,^44,45^ and conditions of chronic pain.^46,47^ Thus, measurement of epifascial and subfascial fat deposits may also be of value for patients with chronic lymphedema and for the understanding of the underlying pathophysiological processes in fat deposition.

In this study, we did not investigate specific fat/water distribution patterns at a cellular level in the subfascial compartment (i.e., between muscle cells or within muscle cells) neither any specific patterns in the epifascial compartment. Also we cannot conclusively identify any pathophysiological effects related to changes in fat and water volumes in lymphedema. The understanding of possible fat distribution-mediated effects and the molecular mechanisms that regulate different pathophysiological conditions (i.e., insulin sensitivity, chronic pain, and proinflammatory actions) in lymphedema need to be further investigated.

### Limitations

The study population was relatively small, including seven patients with arm lymphedema and six with leg lymphedema, thus statistical analysis was performed without differentiating between arm and leg lymphedema. Consequently, we cannot exclude that outcomes might be different for arms and legs. Also, only three cross-sections of 5 mm each (in total 15 mm) were investigated in each limb, which may not be representative for the whole limb. Water–fat MRI cannot differentiate water signals between fluid and muscle, which makes it difficult to evaluate subfascial tissue changes. Regarding controls, these were not age matched, but since each control individual is its own control (right and left arm), this may not have a significant impact on the outcome.

## Conclusion

Using water–fat MRI-based fat quantification; the fat and water contents may be quantified and localized in the various compartments in the lymphedematous limb.

This study shows that the excess volume of epifascial fat and water decreased after liposuction. Also, the excess volume of the lymphedematous limbs was reduced completely when compared with the nonlymphedematous limbs. Interestingly, we also found an excess volume of fat in the subfascial compartment in the lymphedematous limbs that did not change over time. In contrast, the excess volume of water in the subfascial compartment was reduced over time, which may represent a decrease of muscle volume after treatment caused by less mechanical load after liposuction. The water–fat MRI can be used to study the detailed pathophysiological consequences of lymphedema as well as any possible effects after use of anti-inflammatory drugs to decrease fat deposition. It may also be used together with TDC and BIS, which analyzes superficial changes for standardization and also to study changes in deep compartments of the extremities.
